# Evaluation of Magnetic Resonance Imaging for Microsurgical Efficacy and Relapse of Rolandic Meningioma

**DOI:** 10.1155/2022/1026494

**Published:** 2022-06-06

**Authors:** Peng Cao, Nianhua Wang

**Affiliations:** Department of Neurosurgery, Changde First People's Hospital, Changde 415000, Hunan, China

## Abstract

In this study, magnetic resonance imaging (MRI) was used to evaluate the relapse features of patients with Rolandic meningioma after the microsurgery. 53 patients with Rolandic meningioma were selected as the research objects, and they were divided into the relapse group (*n* = 16) and nonrelapse group (*n* = 37) according to whether patients had a relapse during the follow-up period. Differences in quality of life, ^1^H-MRS index, vascular density, and cell proliferation between the two groups were assessed as well as imaging differences between the two groups were analyzed using MRI. The results showed that the patients' quality-of-life scores in the two groups increased notably after the surgical treatment (*P* < 0.05). Compared with the nonrelapse group, the proportion of irregular boundary and uneven enhancement of focal tissue in the relapse group was signally increased (*P* < 0.05). Compared with the nonrelapse group, cell proliferation index, vascular density and imaging index, mean tumor diameter, mean transit time (MTT), time to peak (TTP), fractional anisotropy (FA), choline (Cho)/N-acetylaspartic acid (NAA), Cho/creatine (Cr), lactic acid (Lac)/Cr, and the maximum value of relative cerebral blood volume (rCBVmax) in the relapse group were obviously increased (*P* < 0.05). However, the apparent dispersion coefficient, NAA/Cr, and Lac/NAA values decreased greatly (*P* < 0.05). To sum up, the microsurgical treatment helped improve the quality of life of patients with Rolandic meningioma, and MR imaging could be used to determine the relapse of Rolandic meningioma after microsurgical treatment.

## 1. Introduction

Meningioma is a very common intracranial tumor that originates from arachnoid cells and is only less common than glioma [1]. Meningioma can occur anywhere in the brain. The most common sites are the parasagittal sinus, the convex surface of the brain, and the cerebral falx [2]. Surgical resection and postoperative radiotherapy and chemotherapy are the main methods for the treatment of meningioma, but surgical resection can only control the tumor locally. Besides, the progression of the tumor is closely related to the degree and extent of resection [3, 4]. According to some explorations, the relapse rate of patients with meningioma after surgical treatment is over 20% [5]. The relapse rate and disability rate of patients with Rolandic meningioma are much higher than those of patients with convex meningioma [6]. The Rolandic area has a central system that controls body movement and sensation, and the blood supply of meningioma near the central functional area is abundant [7]. Therefore, meningioma in the Rolandic area is more closely attached to meninges or veins. The tumor separation and resection along the tumor separation interface can damage the cortex of the Rolandic area, which causes temporary or permanent paralysis, severe brain edema, and coma or death after surgery [8]. In the surgical treatment of patients with Rolandic meningioma, the functional area cortex and central sulcus vein need to be protected.

According to the guidelines of the European Society of Neuro-Oncology, the main diagnostic method for meningioma is magnetic resonance imaging (MRI). At present, the main method of imaging assessment of meningioma invasion of brain tissue is also MRI. Magnetic resonance examination is characterized by multidirectional and external parametric imaging, which can clearly show the lesions of tumor soft tissue and microvessels [9]. Magnetic resonance examination includes magnetic resonance imaging (MRI), MRI enhancement imaging, MRI functional imaging, MR diffusion imaging, MR perfusion imaging, and proton magnetic resonance spectroscopy (^1^H-MRS) [10]. Then, features of Brownian movement of brain water molecules, brain cell injury, local dynamic cerebral blood perfusion, tissue microvascular distribution, and biochemical metabolism at the lesion site are all demonstrated noninvasively [11, 12]. Consequently, MR imaging technology has been widely applied in the evaluation of postoperative tumor relapse in patients who receive neurosurgery operations such as meningioma.

In order to investigate the value of MRI in evaluating the efficacy of microsurgical treatment of patients with meningioma, patients with Rolandic meningioma treated by microsurgery were selected as the study subjects. According to the prognosis, follow-up observation was performed, and the patients were divided into relapse group and nonrelapse group. MRI was used to evaluate the imaging features of postoperative recurrence in patients with meningioma, providing a theoretical basis for the diagnosis, treatment, and prognosis evaluation of meningioma in the future.

## 2. Methods

### 2.1. The Research Objects

Fifty-three patients with Rolandic meningioma who were admitted to the hospital from January 2019 to December 2021 were selected as the subjects. The age ranged from 21 to 73 years old, with an average of (53.8 ± 4.4) years old. There were 15 male patients and 38 female patients. The patients were divided into relapse group (*n* = 16) and nonrelapse group (*n* = 37) according to whether they relapsed during the follow-up period. The postoperative recurrence was evaluated by MRI. Recurrent meningioma was defined as the occurrence of new tumors in the surgical site after total resection and the recurrence of clinical symptoms in patients with incomplete resection. This study was approved by the ethics committee of the hospital, and all the enrolled patients were informed and consented.

The inclusion criteria were as follows: (i) patients who were diagnosed with Rolandic meningioma by the preoperative imaging examinations, (ii) patients with the first episode, (iii) patients with meningioma confirmed by the postoperative pathology, (iv) patients who received the craniotomy for microsurgery, (v) patients without the diseases of other vital organs except for meningioma, and (vi) patients with complete clinical data. The exclusion criteria were as follows: (i) patient with multiple intracranial meningiomas; (ii) patients with other types of intracranial neoplasms; (iii) patients who received chemotherapy or radiotherapy before surgery; (iv) patients with diabetes, hypertension, cardiovascular disease, severe organ failure, and other chronic diseases; (v) patients with contraindications to the magnetic resonance examination; (vi) patients who were allergic to contrast agents; and (vii) patients in pregnancy or lactation.

### 2.2. The Surgical Methods

Total resection of Rolandic meningioma was performed on the premise of protecting important structures such as the functional cortex and reflux veins. Imaging techniques such as MRI and computed tomography (CT) were used to locate the position of the tumor before surgery. Then, the tumor was gently removed under a microscope during surgery. For the tumors with a maximum diameter of less than 3 cm, blood supply needed to be blocked firstly. Subsequently, the tumor was separated gradually along with the arachnoid space and resected. If the tumor was closely connected to the surrounding tissues and could not be separated, a small amount of tumor tissue could remain. When preoperative MRI showed there was high intracranial pressure or obvious edema around the tumor, the blood supply around the tumor needed to be blocked firstly, and the tumor was separated and removed repeatedly. Alternatively, the tumor was removed with an ultrasonic suction device and intracapsular decompression. In the process of tumor separation, proximity to the tumor is necessary to avoid vascular injury. The patients were monitored in the intensive care unit (ICU) for 24 hours postoperatively, and the imaging review was performed within 48 hours postoperatively. Accordingly, appropriate mannitol and glucocorticoid therapy were given to the patients with severe edema.

### 2.3. Magnetic Resonance Scanning

3.0 T magnetic resonance scanner was used to scan the whole brain region. The scanning sequence included T1-weighted imaging (T1WI) plain scan, T1WI enhancement, T2-weighted imaging (T2WI), diffusion-weighted imaging (DWI), perfusion-weighted imaging (PWI), and single voxel PRESS.

The scan parameters of sagittal T1WI were set as the repetition time was 16 ms, and the echo time was 400 ms. The scanning parameters of axial T1WI were set as the repetition time was 12 ms, and the echo time was 400 ms. The scanning parameters of axial T2WI were set as the repetition time was 80 ms, and the echo time was 4,000 ms. The scanning parameters of gradient-echo echo-planar imaging (GRE-EPI) for T2WI were set as follows: the field size was 240 *∗* 240 mm^2^, the matrix size was 256 *∗* 256, the repetition time was 2,000 ms, the echo time was 80 ms, the layer thickness was 5 mm, the layer spacing was 0 mm, and the flip angle was 90°.

The scanning parameters of DWI were set as follows: the field size was 230 *∗* 230 mm^2^, the matrix size was 512 *∗* 512, the repetition time was 100 ms, the echo time was 6,000 ms, the layer thickness was 6 mm, the layer number was 16, the layer spacing was 0.35 mm, and the *b* value was 1,000 s/m.

Then, the 18 G vein needle was connected to the patients' cubital vein and fixed. After a special MR syringe was used for connection and positioning, the 0.2 mmol/kg gadopentetate dimeglumine was injected at a speed of 4 mL/s. The original perfusion image was obtained by injecting 20 mL 0.9% NaCl at the same speed and imaging for 1.5 min.

### 2.4. Observation Indexes

For the quality of life, the quality-of-life scale for cancer patients enacted by the World Health Organization was used to score the different dimensions of quality of life for patients 1 year after surgical treatment. The evaluation items included physical function, cognitive function, role function, emotional ability, and social function. The higher the score was, the better the patient's quality of life was.

For the MR perfusion indexes, the Functool was employed for the construction of the pseudocolor image of the organizational interface from the obtained original data. The maximum perfusion region in tumor parenchyma was set as the region of interest. The mean value of cerebral blood volume (CBV) was calculated after the 5 tests, and the ultimate maximum value of relative cerebral blood volume (rCBVmax) of patients was calculated.

For the DWI indexes, the time to peak (TTP), mean transit time (MTT), apparent dispersion coefficient (ADC), and fractional anisotropy (FA) were detected automatically by the Siemens workstation.

For the ^1^H-MRS indexes, the peak values of N-acetylaspartic acid (NAA), choline (Cho), creatine (Cr), lactic acid (Lac), and lipids (Lip) were observed, and the indexes of NAA/Cr, Cho/Cr, Cho/NAA, Lac/Cr, and Lac/NAA were calculated.

### 2.5. Observation Methods of Vascular Density and Cell Proliferation

For vascular density and cell proliferation, the lesion tissues were removed by surgery, which was fixed with 10% formaldehyde solution and paraffin, and paraffin sections were made. Subsequently, immunohistochemical staining and eosin redyeing were used for the sections. Polyclonal endothelial cell marker antibody, antiproliferative cell antibody, and antigen-antibody were used for the marker detection. A light microscope was used to observe the tissue staining after it was cultured at room temperature for 1 h. The positive capillaries and tumor nuclei were counted from 10 visions, and the mean value was obtained to calculate the vascular density (VD) and cell proliferation index (CPI).

### 2.6. Statistical Methods

SPSS 22.0 was used for statistical analysis. Mean ± standard deviation ($\bar{x}$ ± *s*) was how quantitative data were expressed, and the independent sample *t* test was used to compare the differences between the groups. Percentage (%) was how count data were expressed, and the *χ*^2^ test was used to compare differences between the groups. The test level was expressed as *α* = 0.05. The difference was statistically significant with *P* < 0.05.

## 3. Results

### 3.1. Comparison of Basic Clinical Features of Patients

The differences in basic clinical features of patients were compared between the relapse group and the nonrelapse group. In Table 1, there were insignificant differences in the mean age, sex ratio, and tumor weight between the two groups (*P* > 0.05). The proportion of partial resection in the relapse group was manifestly higher than that in the nonrelapse group (*P* < 0.05).

### 3.2. Comparison of Quality of Life between the Two Groups

**Figure 1:**
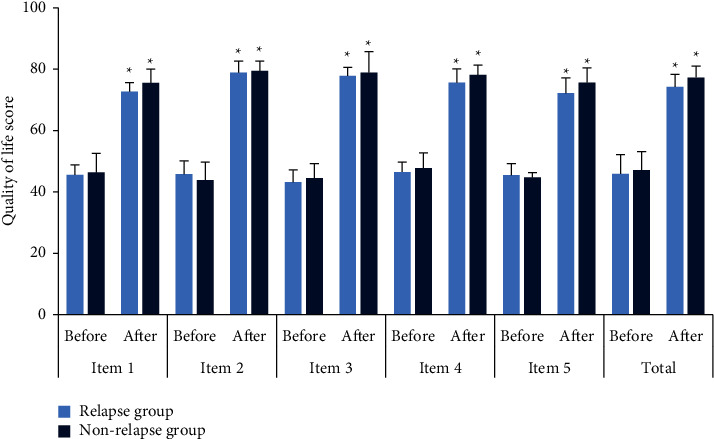
Quality-of-life scores in each group before and after the surgery. Item 1: physical function; item 2: cognitive function; item 3: role function; item 4: emotional ability; item 5: social function; ^*∗*^compared with the scores before the surgery, *P* < 0.05.

The difference in the quality-of-life scores before and after surgical treatment was compared between the two groups. In Figure 1, compared with the conditions before surgery, the physical function, cognitive function, role function, emotional ability, social function, and total scores of patients in the relapse group and the nonrelapse group were remarkably increased after treatment (*P* < 0.05). There were no considerable differences in each item's quality-of-life scores between the two groups before and after surgery (*P* > 0.05).

### 3.3. Comparison of Histopathological Features of Patients

**Figure 2:**
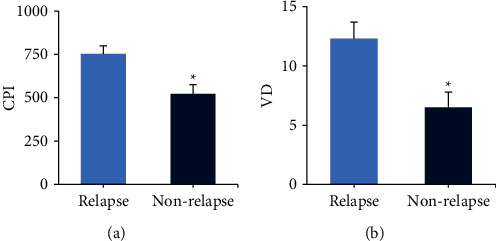
Cell proliferation index and vascular density in each group. (a) Cell proliferation index; (b) vascular density; ^*∗*^compared with the relapse group, *P* < 0.05.

The differences in CPI and VD of patients were compared between the relapse group and the nonrelapse group. In Figure 2, CPI was (753.4 ± 45.6) and VD was (12.3 ± 1.4) in the relapse group. In the nonrelapse group, CPI was (522.8 ± 53.1) and VD was (6.5 ± 1.3). The CPI and VD values in the relapse group were notably lower than those in the nonrelapse group (*P* < 0.05).

### 3.4. Comparison of MR Imaging Features of Patients

**Figure 3:**
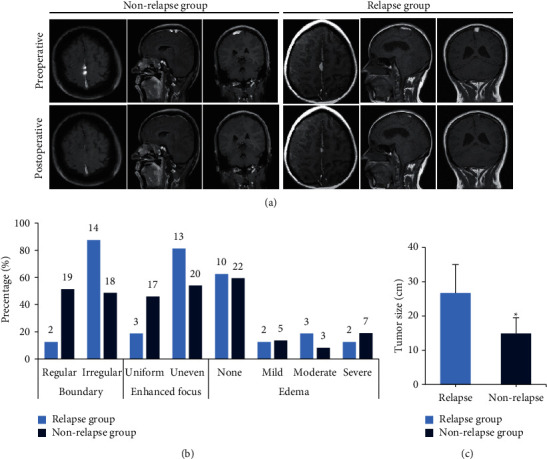
MRI features of patients in each group. (a) Preoperative and postoperative MRI imaging; (b) basic features of MRI; (c) tumor size. ^*∗*^Compared with the relapse group, *P* < 0.05.

In Figure 3, the basic MRI features and MRI images before and after surgery were compared between the two groups. In the nonrelapse group, the lesion tissue was completely resected. In the recurrence group, part of the tumor tissue was preserved because the tumor was closely connected to the surrounding tissues (Figure 3(a)). The basic features of MRI were compared between the two groups. The mean tumor diameter of the relapse group and the nonrelapse group was (26.7 ± 8.3) cm and (14.9 ± 4.6) cm, respectively, with significant differences between the two groups (*P* < 0.05) (Figures 3(b) and 3(c)). The proportion of lesion tissues with irregular boundary and nonuniform enhancement in the relapse group was evidently higher than that in the nonrelapse group (*P* < 0.05). There were insignificant differences in the proportion of edema degree between the two groups (*P* > 0.05).

**Figure 4:**
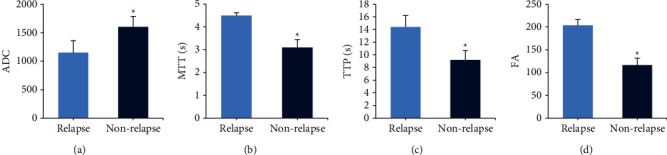
MR-DWI sequence imaging features of patients in each group. (a) Apparent dispersion coefficient; (b) mean transit time; (c) time to peak; (d) fractional anisotropy; ^*∗*^compared with the relapse group, *P* < 0.05.

The differences in the related parameters of DWI were compared between the two groups. In Figure 4, the ADC values of patients in the relapse group and nonrelapse group were (1150.73 ± 210.92) and (1604.32 ± 182.10), respectively, with a considerable difference between the groups (*P* < 0.05). The MTT values in the relapse and the nonrelapse groups were (4.51 ± 0.12)s and (3.11 ± 0.34)s, respectively, and there were significant differences between the groups (*P* < 0.05). The TTP values in the relapse group and the nonrelapse group were (14.40 ± 1.82)s and (9.22 ± 1.50)s, respectively, and the difference was significant in the two groups (*P* < 0.05). The FA values in the relapse group and the nonrelapse group were (203.73 ± 12.54) and (116.33 ± 15.16), respectively, with a considerable difference in the two groups (*P* < 0.05).

**Figure 5:**
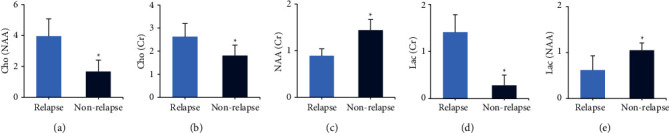
MR-^1^H-MRS sequence imaging features of patients in each group. (a) Cho/NAA values; (b) Cho/Cr value; (c) NAA/Cr value; (d) Lac/Cr value; (e) Lac/NAA value. ^*∗*^Compared with the relapse group, *P* < 0.05.

The differences in the parameters of the ^1^H-MRS sequence were compared between the two groups. In Figure 5, the Cho/NAA values of patients in the relapse group and nonrelapse group were (3.95 ± 1.12) and (1.67 ± 0.74), respectively, and the difference was considerable between the groups (*P* < 0.05). Cho/Cr value was (2.63 ± 0.57) in the relapse group and that was (1.81 ± 0.45) in the nonrelapse group, and there was a significant difference between the two groups (*P* < 0.05). NAA/Cr value was (0.89 ± 0.15) in the relapse group and that was (1.44 ± 0.23) in the nonrelapse group, with a significant difference between the groups (*P* < 0.05). The Lac/Cr values were (1.41 ± 0.37) and (0.28 ± 0.22) in the relapse group and nonrelapse group, respectively, with significant differences between the groups (*P* < 0.05). The Lac/NAA values were (0.62 ± 0.31) and (1.05 ± 0.16) in the relapse group and nonrelapse group, respectively, and the difference was significant in the two groups (*P* < 0.05).

**Figure 6:**
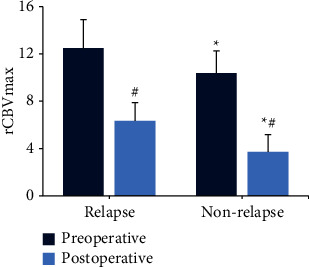
The rCBVmax values in each group. ^*∗*^Compared with the relapse group, *P* < 0.05; ^#^compared with the conditions before the surgery, *P* < 0.05.

The difference in the parameters of MR perfusion was compared between the two groups. In Figure 6, before surgery, the rCBVmax values of the relapse group and the nonrelapse group were (12.48 ± 2.41) and (10.37 ± 1.88), respectively, with significant differences between the groups (*P* < 0.05). The rCBVmax values of patients in the relapse group and the nonrelapse group were (2.41 ± 1.53) and (1.88 ± 1.46), respectively, and the difference between the groups was considerable (*P* < 0.05). Besides, the rCBVmax values in the two groups after the surgery were markedly lower than those before the surgery (*P* < 0.05).

## 4. Discussion

Meningioma is a kind of neuroectodermal tumor that develops from arachnoid cells [13]. For most meningiomas, the arterial blood supply is performed through the anterior and posterior choroid arteries, so the incidence of meningiomas in the lateral ventricle is highest [14]. Meningiomas near the Rolandic area are supplied by both internal and external carotid arteries, so the meningiomas are closely attached to the pia mater of the cortex and the central reflux veins in the functional area. Consequently, surgical separation and removal of tumors can result in cortical or venous reflux in functional areas, thus causing increased rates of recurrence, disability, and mortality [15,16]. MR imaging technology can precisely distinguish the soft tissue and blood vessels, which is often applied in the diagnosis of cerebrovascular diseases [17]. The differences in MR imaging features of patients with Rolandic meningioma after microsurgical treatment were compared between the relapse and the nonrelapse groups.

According to the results, all the 53 patients with Rolandic meningioma successfully received the microsurgical treatment without any death, and the quality-of-life scores of patients 1 year after surgery were observably higher than those before surgery. The quality of life of patients with Rolandic meningioma was manifestly improved after the tumor separation and resection under the microscope. Therefore, this operation has high safety, which is consistent with the results of Zhou et al. [18]. Moreover, the location, size, morphology, heterogeneity, and calcification degree of the tumor could be used to determine tumor relapse [19, 20]. MTT referred to the time for the contrast agent to pass through the capillary network of brain tissue in MR perfusion imaging [21]. TTP referred to the time from the beginning of injection to the peak concentration of the contrast agent [22]. The results of the experiment reflected that the rCBVmax, MTT, and TTP were remarkably increased in the relapse group compared with the nonrelapse group. PWI uses a contrast agent to make the local small magnetic field inhomogeneous to detect changes in blood flow. rCBV value is an important indicator in PWI examination and can reflect the number of tumor blood vessels [23]. rCBV is closely related to the pathological grade of the tumor. The higher the degree of tumor differentiation, the richer the blood supply and the higher the rCBV value [24]. VD can quantitatively evaluate tumor angiogenesis, so it is used in the analysis of malignant tumors and is considered as a marker for prognosis assessment of patients with various cancer types [25]. Hence, for the patients who had the postoperative relapse, the blood flow of brain tumors was abundant, and the number of blood vessels was increased.

^1^H-MRS is a noninvasive method to help explore the biochemical metabolism of brain tumors, which can be used to distinguish the tissue types of brain tumors and the lesion areas of the lung tumors and the tumors [26]. According to the results, compared with the nonrelapse group, Cho/NAA, Cho/Cr, and Lac/Cr values in the relapse group were evidently increased, while the NAA/Cr and Lac/NAA values were decreased. The above results showed that there were substantial changes in the biochemical metabolism of patients with relapse of meningioma, and the increase in Cho and the decrease in NAA were all caused by the decrease in the number of neuron cells [27]. When the brain tumor relapses, the diffusion rate of cellular water molecules in the tumor tissue is obviously slowed down, and the MRI diffusion imaging shows high signal characteristics [28]. Both ADC and FA values are used to evaluate the integrity of brain white matter fiber bundles and myelin sheath [29]. Moreover, the ADC value is closely negatively correlated with the tumor cells [30]. Compared with the nonrelapse group, the ADC value was signally increased, and the FA value was obviously decreased in the relapse group, possibly because the damage of brain tissue was caused by brain white matter and tumor edema in patients with recurrent brain tumors, which was similar to the results of Fages et al. [31].

## 5. Conclusion

MRI was used to evaluate the surgical efficacy of patients with meningioma. The results showed that various MR imaging techniques were helpful to accurately and objectively reflect the physiological characteristics and imaging features of the Rolandic meningioma, which was conducive to identifying the relapse factors. It has clinical application value. However, the changes in MR imaging features of patients with relapse and nonrelapse meningiomas near the Rolandic area are only analyzed, and the influencing factors of postoperative relapse of the Rolandic meningioma are not explored deeply. In the future, it is necessary to collect more clinical data on the treatment of meningiomas near the Rolandic area, and comprehensive analysis of the risk factors for tumor relapse after microsurgery is needed.

## Figures and Tables

**Table 1:** Comparison of basic clinical features of patients.

Group	Age (years old)	Male/female (*n*)	Methods of resection (*n* (%))		Tumor weight (g)	Time torelapse (years)
			Total resection	Partial resection		
Relapse group (*n* = 16)	48.9 ± 3.6	5/11	2 (12.5)	14 (87.5)	46.3 ± 8.5	2.7 ± 0.5
Nonrelapse group (*n* = 37)	49.7 ± 2.8	10/27	22 (59.5)	15 (40.5)	40.1 ± 9.6	—
Statistical value	−0.747	0.148	−3.998		0.562	—
*P*	0.402	0.233	0.005		0.121	—

*Note.* The statistical values included the values of *t* and *χ*^2^.

## Data Availability

The data used to support the findings of this study are available from the corresponding author upon request.
